# A Targeted “Capture” and “Removal” Scavenger toward Multiple Pollutants for Water Remediation based on Molecular Recognition

**DOI:** 10.1002/advs.201500289

**Published:** 2015-12-10

**Authors:** Jie Wang, Haijing Shen, Xiaoxia Hu, Yan Li, Zhihao Li, Jinfan Xu, Xiufeng Song, Haibo Zeng, Quan Yuan

**Affiliations:** ^1^ Key Laboratory of Analytical Chemistry for Biology and Medicine (Ministry of Education)College of Chemistry and Molecular SciencesWuhan UniversityWuhan430072P.R. China; ^2^ Institute of Optoelectronics and NanomaterialsSchool of Materials Science and EngineeringNanjing University of Science and TechnologyNanjing210094P.R. China

**Keywords:** adsorbent, aptamer, hydrogel, Janus nanoparticle, water treatment

## Abstract

For the water remediation techniques based on adsorption, the long‐standing contradictories between selectivity and multiple adsorbability, as well as between affinity and recyclability, have put it on weak defense amid more and more severe environment crisis. Here, a pollutant‐targeting hydrogel scavenger is reported for water remediation with both high selectivity and multiple adsorbability for several pollutants, and with strong affinity and good recyclability through rationally integrating the advantages of multiple functional materials. In the scavenger, aptamers fold into binding pockets to accommodate the molecular structure of pollutants to afford perfect selectivity, and Janus nanoparticles with antibacterial function as well as anisotropic surfaces to immobilize multiple aptamers allow for simultaneously handling different kinds of pollutants. The scavenger exhibits high efficiencies in removing pollutants from water and it can be easily recycled for many times without significant loss of loading capacities. Moreover, the residual concentrations of each contaminant are well below the drinking water standards. Thermodynamic behavior of the adsorption process is investigated and the rate‐controlling process is determined. Furthermore, a point of use device is constructed and it displays high efficiency in removing pollutants from environmental water. The scavenger exhibits great promise to be applied in the next generation of water purification systems.

## 1 Introduction

Providing safe drinking water for human survival and development remains an overriding priority of the 21st century because waterborne diseases are still one of the leading causes of death in the world.^1^ Besides, it was estimated that the rapid growth of population and serious water pollution are going to put about three billion people below the water stress threshold by 2025.^2^ Consequently, water purification has already become a necessity in nearly all of regions to afford safe drinking water. Among all of the existing techniques developed over the past years for water treatment, adsorption has been demonstrated to be one of the most effective and convenient approaches due to its simple operation and energy effectiveness.^3^ Usually, ideal adsorbents should possess the following features: perfect selectivity,^4^ multiple adsorbability,^5^ strong affinity,[[qv: 3c]],^6^ good reusability,^5^, ^6^, ^7^ no secondary pollution,^5^, ^8^ rapid kinetics,^9^ and high adsorption capacity.[[qv: 3c]] Although many adsorbents have been created, the ideal adsorbent is still to be discovered since some of these features are internally contradictory. For instance, adsorbents with perfect selectivity can only remove limited kinds of contaminants and thus cannot deal with real water samples;^10^ strong binding affinity usually causes an ineffective desorption process and thus leads to poor reusability.^11^ As a consequence, it is still challenging to simultaneously fulfill all of these requirements for ideal adsorbents.

The aforementioned seven features are hard to be satisfied by individual material. Hybrid, which means to integrate different components at nanoscale to make a single object with enhanced properties, has become one of the most popular research areas in materials science.^12^ Lego‐like chemistry is one of the mostly well‐developed hybridization methods and it allows fine control of hybrid materials' structure and properties on the semilocal scale through manipulating each preformed hybrid component in a step‐by‐step manner.^13^ Therefore, Lego‐like chemistry may serve as a promising method to develop composite materials for meeting the features of ideal adsorbents.

Herein, by using Lego‐like chemistry, we have created a novel adsorbent scavenger through rationally combining the advantages of anisotropic Janus‐type hybrid nanoparticles,^14^ selective recognition capabilities of aptamers, and 3D porous structure of hydrogel in a stepwise manner. This scavenger well satisfies the above‐mentioned features of the ideal adsorbents by overcoming the contradiction between selectivity and multiple adsorbability, as well as the contradiction between binding affinity and reusability. The three main kinds of pollutants in water (heavy metal ions, organic pollutants, and pathogens) can be simultaneously reduced to levels lower than drinking water standards. The thermodynamic features and the rate‐determining steps of the adsorption process were investigated. A point of use (POU) device was further created and it can effectively reduce trace amounts of pollutants to levels below the mandated guidelines for drinking water. The scavenger displayed great promise to be applied to pollutants removal from aqueous solutions in reality. Moreover, this strategy may serve as a universal method for constructing functional hybrid materials with qualitatively and quantitatively improved properties to remedy challenges in other applied research areas.

This is an open access article under the terms of the Creative Commons Attribution License, which permits use, distribution and reproduction in any medium, provided the original work is properly cited.

## 2 Results and Discussion

### 2.1 Construction and Characterization of Aptamers‐Based Scavenger

**Figure 1:**
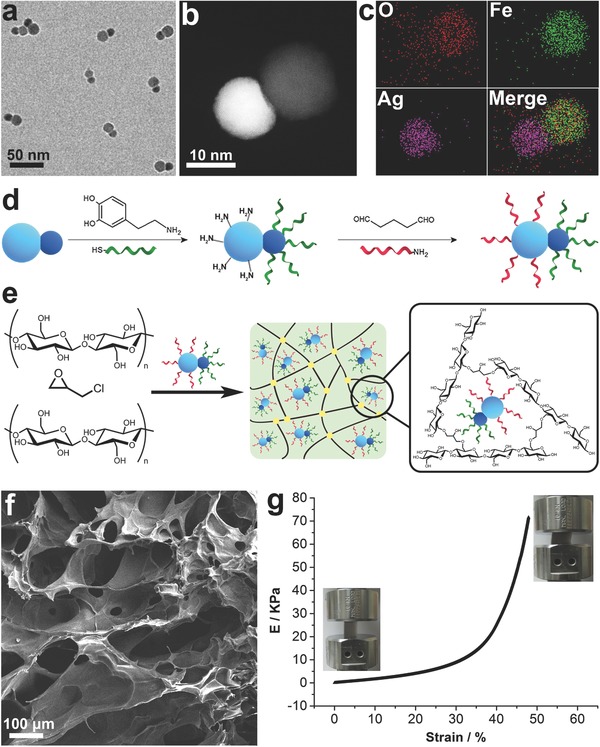
a) TEM, b) HAADF‐STEM, and c) elemental mapping images of Fe_3_O_4_–Ag nanoparticles. d) Schematic illustration of the strategy to prepare the bifunctionalized Janus nanoparticles. e) Schematic representation of the construction of hydrogel scavenger. f) SEM images of the cross‐section of the hydrogel scavenger. g) Stress–strain curves of hydrogel scavenger. Inset: photographs of the scavenger before and after compression.

A two‐step strategy was employed to prepare the composite scavenger, as presented in **Figure** **1**. Transmission electron microscopy (TEM), high‐angle annular dark‐field scanning transmission electron microscopy (HAADF‐STEM), and energy‐dispersive X‐ray analysis were adopted to characterize the Fe_3_O_4_–Ag Janus nanoparticles. As shown in Figure 1a and Figure S2 (Supporting Information), the nanoparticles were monodispersed with average sizes of Fe_3_O_4_ at about 15 nm and Ag at about 10 nm, respectively. HAADF‐STEM (Figure 1b), elemental mapping (Figure 1c), and line scan analysis (Figure S4, Supporting Information) clearly demonstrated the well‐defined dumbbell‐shaped structures of Fe_3_O_4_–Ag Janus nanoparticles. The Ag surface was further functionalized with Hg^2+^‐binding aptamers,^15^ and Fe_3_O_4_ surface with BPA‐binding aptamers (Figure 1d).^16^ The successful functionalization of Janus nanoparticle by aptamers was verified by zeta potential measurement (Figure S9, Supporting Information). Figure 1e showed that the final composite scavenger was prepared by entrapping aptamers‐functionalized Janus nanoparticles into cellulose hydrogel.^17^ This porous composite sorbent can efficiently eliminate secondary pollution associated with uncontrolled nanoparticle dispersions since it held the nanoparticles and kept the loaded contaminants securely within a stable structure.[[qv: 7a]] Scanning electron microscope was utilized to investigate the structure of the hydrogel scavenger (Figure 1f). As expected, a highly open porous structure was observed, which could provide sufficient space for water and thus allowing for easy diffusion of pollutants to the binding sites. Stress–strain curve (Figure 1g and Figure S14, Supporting Information) suggested that the scavenger can be compressed up to 50% without rupture, showing its good mechanical strength to withstand relatively high flow rates when it was applied to infrastructure such as permanent membrane filter.[[qv: 7a]]

### 2.2 Selectivity and Multiple Adsorbability Tests

**Figure 2:**
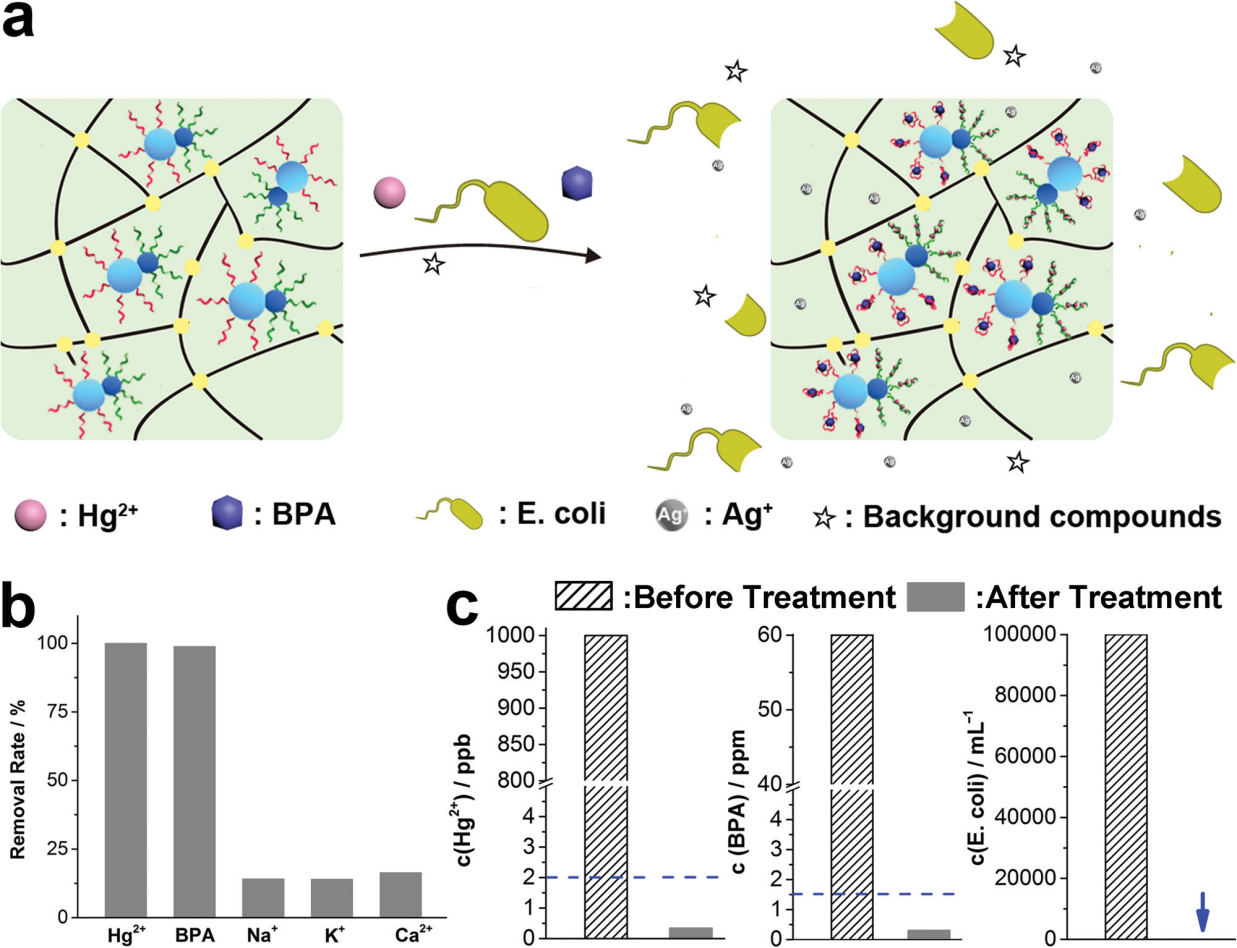
a) Schematic illustration of selectively removing multifold pollutants simultaneously. b) Removal efficiencies of the scavenger toward different substances in selectively tests. c) Concentrations of Hg^2+^, BPA, and *E. coli* before and after treatment. Dotted line: the upper limits for corresponding pollutants in drinking water. Arrow: no bacterial growth was observed.

Unlike traditional adsorbents, this scavenger was capable of selectively removing multiple pollutants from water simultaneously. As illustrated in **Figure** **2**a, when exposed to target pollutants and background elements, aptamers in the scavenger folded into special binding pocket structures to specifically bind with their corresponding targets, while background elements were left undisturbed. In addition, silver ions dissolved in the aqueous solution from the scavenger can effectively kill pathogens. This property was verified by investigating the selectivity and multiple adsorbability of the scavenger. Selectivity test was performed by capturing Hg^2+^ and BPA in the presence of Na^+^, K^+^, Ca^2+^, and so on. As shown in Figure 2b and Figure S15 (Supporting Information), the scavenger effectively removed Hg^2+^ and BPA but adsorbed little of background elements, which was attributed to the excellent selective recognition abilities of aptamers.^18^ Furthermore, the multiple adsorbability of this scavenger was examined by treating water containing Hg^2+^ (1 ppm), BPA (60 ppm), and *Escherichia coli* (≈10^5^ mL^−1^). As shown in Figure 2c, these three kinds of pollutants can be effectively removed by the same scavenger with high efficiencies (99.96% for Hg^2+^, 98.9% for BPA, and 100% for *E. coli*), clearly suggesting the multiple adsorbability of the scavenger for dealing with different kinds of pollutants. It was noteworthy that the residual concentration of Hg^2+^ in treated water was about 0.34 ppb, much lower than the upper limit of 2 ppb mandated by the United States Environmental Protection Agency (EPA); concentration of BPA was about 0.66 ppm, adhering to the estimated guideline upper value of 1.5 ppm for BPA in drinking water. These results therefore clearly demonstrated that this scavenger overcame the contradiction between selectivity and multiple adsorbability, which were attributed to the synergistic effect elicited by the anisotropic features of Janus nanoparticles and the selective recognition function of aptamers.

### 2.3 Binding Affinity Determination and Reusability Tests

Next, the binding affinity and reusability of the scavenger were investigated to test whether these contradictory features could also be satisfied. The binding affinity of the scavenger toward Hg^2+^ and BPA was determined by measuring distribution coefficient (*K*_d_) based on removal efficiencies in the above selectivity tests.^19^ *K*_d_ is an important metric to evaluate any sorbent's performance and it was defined as

(1) $$K_{d}=\frac{C_{i}-C_{f}}{C_{f}}\times \frac{V}{m}$$

where *C*_i_ was the original concentrations of pollutants, *C*_f_ was the concentrations at equilibrium, *V* was the volume of water sample (mL) treated, and *m* was the mass of adsorbents used (g). According to previous studies, *K*_d_ values above 10^3^ mL g^−1^ were very good and those above 10^4^ mL g^−1^ were considered to be excellent.^20^ The *K*_d_ values of this scavenger toward Hg^2+^ and BPA were calculated to be 1.24 × 10^6^ and 9.72 × 10^4^ mL g^−1^, respectively. These results suggested that this scavenger possessed exceptional high affinity to pollutants, which may be ascribed to the strong binding affinity of aptamers toward their targets.^21^

**Figure 3:**
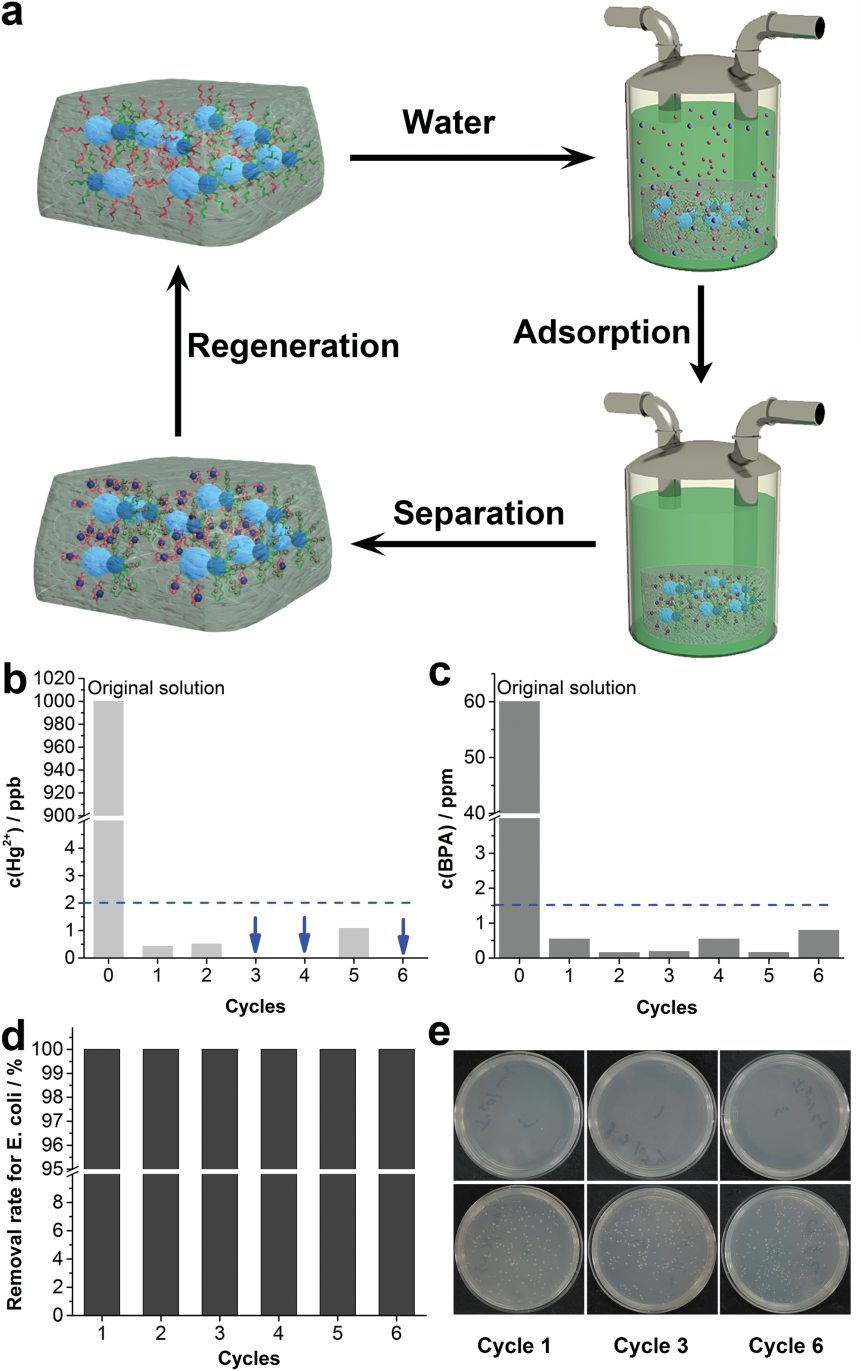
a) Schematic representation of cyclic removal of multiple pollutants. Concentrations of b) Hg^2+^ and c) BPA in the tested cycles. Dotted line: the upper limits for corresponding pollutants in drinking water. Arrow: concentrations of Hg^2+^ lower than the detection limit of atomic fluorescence spectrometry. d) Removal efficiencies of *E. coli* in six cycles. e) *E. coli* culturing results of the treated water in cycles 1, 3, and 6. The upper row was the treated water and the lower row was the control samples.

The reusability of this scavenger was investigated by using it for cyclical removal of the above three pollutants. As shown in **Figure** **3**a, aptamers specifically sequestered the targets by folding into unique binding pockets. The bulk sorbent with the loaded pollutants can be easily separated from the solution and thus purified water was obtained. The isolated scavenger was regenerated by denaturizing aptamers to release the loaded pollutants and it was further applied to the next cycle. As presented in Figure 3b, the residual Hg^2+^ in the treated water was lower than 1.1 ppb in all of the six cycles, that is, above 99.89% of Hg^2+^ can be removed by this scavenger. As for BPA, the residual concentrations in the tested cycles were lower than 0.8 ppm, suggesting that this scavenger can be easily regenerated without significant loss of adsorption efficiency (Figure 3c). This easy regeneration property came from the fact that the binding pockets structure of aptamers could be effectively denatured by means of rising temperature or changing pH values.^22^ Obviously, the rationally designed scavenger perfectly remedied the contradiction between binding affinity and reusability. Accordingly, one can safely draw the conclusion that the main challenges associated with traditional adsorbents were conquered by this novel hydrogel scavenger. In addition, the scavenger was also used to treat water samples containing *E. coli* (≈10^5^ CFU mL^−1^) for several cycles and the disinfection efficiency was shown in Figure 3d. Nearly all of the *E. coli* were killed by the hybrid hydrogel in the tested six cycles (logarithmic removal rate >4) and the antibacterial performance was better than that of some recently developed antibacterial materials.^23^ Photographs of the cultured agar plates were also shown in Figure 3e (cycles 1, 3, and 6). As expected, the treated water samples showed essentially no bacterial growth. In contrast, extensive growth of *E. coli* was observed for water samples before treatment. These results therefore clearly demonstrated the strong binding affinity and good reusability of the composite scavenger, which indicated its promise for water purification in reality.

### 2.4 Secondary Pollution Tests

**Figure 4:**
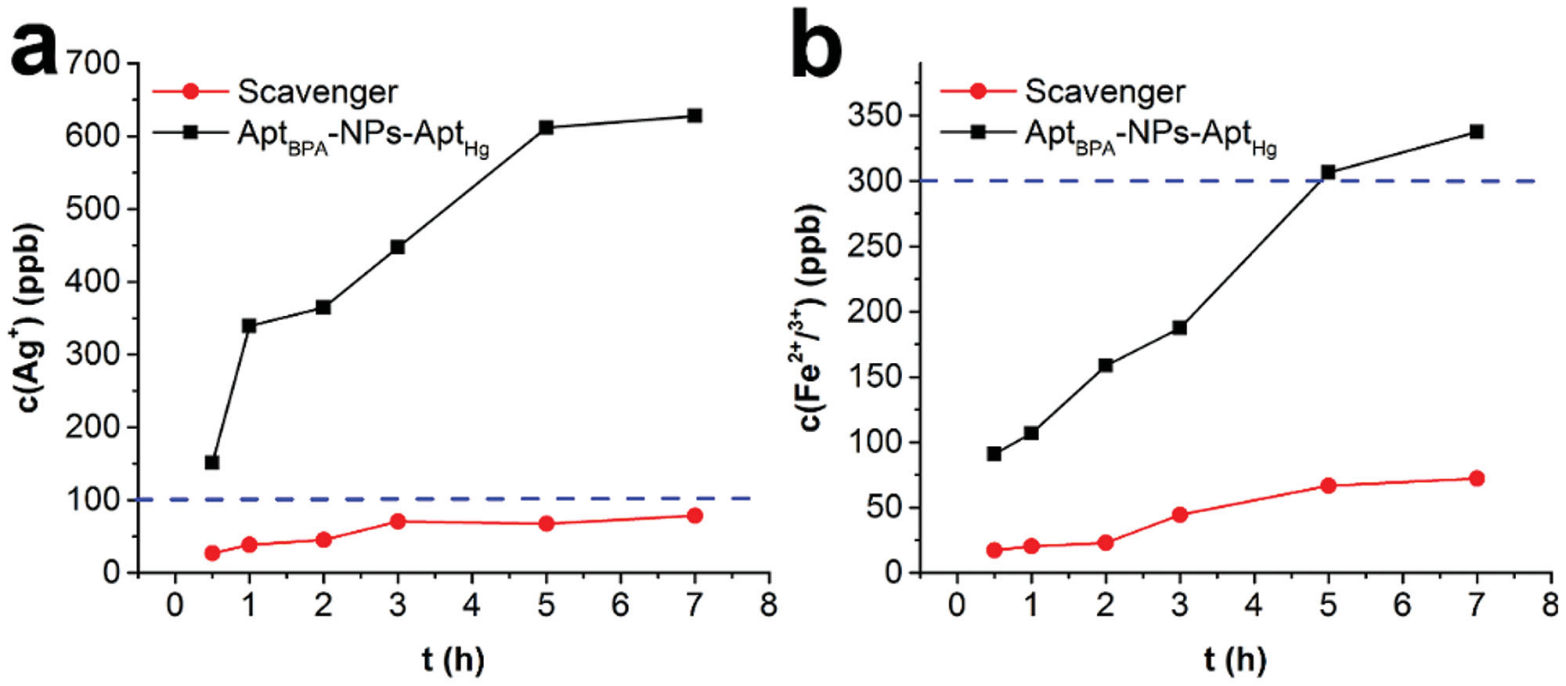
Time evolution of a) the release of silver and b) ferrous/ferric ions from composite scavenger and aptamers‐functionalized Fe_3_O_4_–Ag Janus nanoparticles (designated as Apt_BPA_‐NPs‐Apt_Hg_). Dotted line: the upper limits for corresponding ions in drinking water.

To investigate whether the porous cellulose hydrogel could prevent secondary pollution associated with uncontrolled nanoparticle dispersions, the time evolution of the release of silver and ferrous/ferric ions into water from both the composite scavenger and aptamers‐functionalized Fe_3_O_4_–Ag Janus nanoparticles was monitored. As shown in **Figure** **4**, concentrations of silver and ferrous/ferric ions leached from the scavenger increased first and finally reached the plateau at about 3 and 5 h, respectively. More importantly, the final concentration of silver ions was around 78.6 ppb, lower than the permissible limit of 100 ppb set by the EPA.^24^ Besides, final concentration of ferrous/ferric ions was about 70.2 ppb and this value was also much lower than the upper norms of 300 ppb in drinking water (WHO).^25^ On the contrary, the released silver and ferrous/ferric ions from aptamers‐functionalized Fe_3_O_4_–Ag Janus nanoparticles were much higher than that from composite scavenger. Moreover, the concentrations of silver and ferrous/ferric ions quickly exceeded the permissible limits set by the EPA and WHO, respectively. These results therefore clearly showed that this bulk composite scavenger would not cause secondary pollution, indicating the excellent performance of the porous cellulose polymer in preventing Fe_3_O_4_–Ag Janus nanoparticles from leaching into the environment.

### 2.5 Kinetics and Equilibrium Adsorption Isotherm Studies

**Figure 5:**
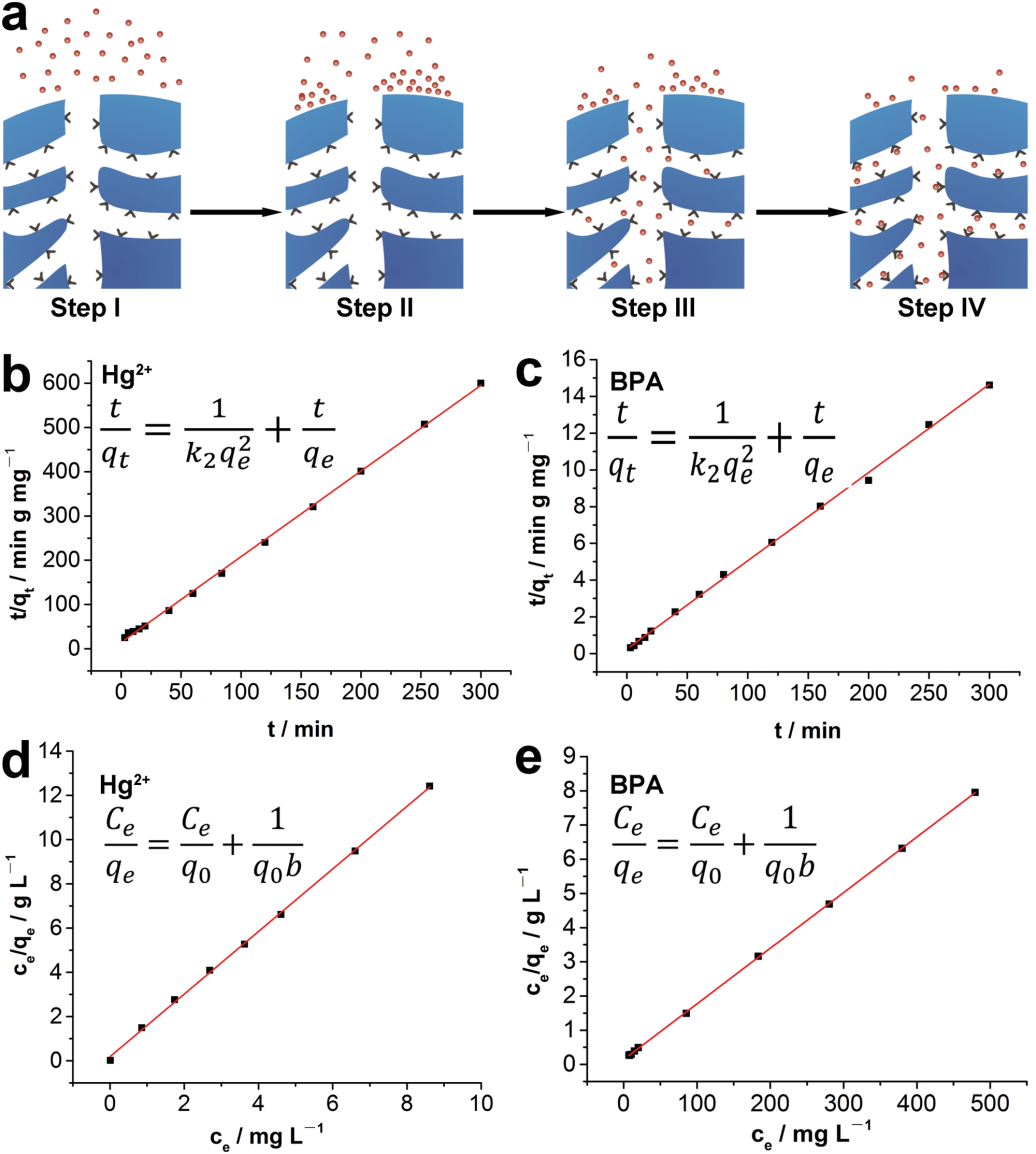
a) Schematic illustration of the four basic steps in the adsorption process. b,c) Pseudo‐second‐order kinetic plots for the adsorption of Hg^2+^ and BPA. d,e) Linear regression for the adsorption of Hg^2+^ and BPA after fitting the equilibrium adsorption data with the Langmuir adsorption model.

To investigate the adsorption rates and loading capacities of the hydrogel scavenger, we performed kinetics and equilibrium adsorption isotherm studies. The four basic steps of adsorption process was illustrated in **Figure** **5**a. Pollutants transported in the bulk solution first (Step I) and then diffused across the liquid film surrounding the scavenger (Step II).^19^ The pollutants further diffused in the pores of the scavenger (Step III) and were finally adsorbed on the binding sites within the porous scavenger (Step IV). Considering the reliability to represent the kinetics for the adsorption of pollutants onto sorbents, the kinetic results were fitted with the pseudo‐second‐order kinetic model with the following equation

(2) $$\frac{t}{q_{t}}=\frac{1}{k_{2}q_{e}^{2}}+\frac{t}{q_{e}}$$

where *k*_2_ (g mg^−1^ min^−1^) was the rate constant, *q*_t_ (mg g^−1^) was the amount of pollutants adsorbed at given time, and *q*_e_ (mg g^−1^) was the amount of adsorbed pollutants at equilibrium. The adsorption kinetics of Hg^2+^ on scavenger was shown in Figure 5b. A high correlation coefficient was obtained by fitting the data to pseudo‐second‐order kinetic model (0.9991), indicating that the adsorption of Hg^2+^ mainly took place on localized sites and the desorption rate of Hg^2+^ was negligible compared to the adsorption rate.^19^ From the above‐fitted line, the value of the adsorption rate constant was determined to be 0.26 g mg^−1^ min^−1^. Such rapid adsorption kinetics can be ascribed to the large open pore structure of hydrogel scavenger to facilitate the diffusion of Hg^2+^ and its large surface area covered with bifunctionalized Janus nanoparticles. Figure 5c represented the adsorption kinetics of BPA and the results were also well fitted to the pseudo‐second‐order kinetic model with correlation coefficient of 0.9987. The adsorption behavior of BPA was similar to that of Hg^2+^ and the adsorption rate constant was determined to be 0.0152 g mg^−1^ min^−1^, larger than that of some previously reported values.^26^ In addition, intraparticle diffusion studies were performed to determine the rate‐controlling steps in the adsorption process, which would provide valuable instructions for the future practical water treatment applications of this hydrogel scavenger. Both the adsorption behavior of Hg^2+^ and BPA were well fitted with the equation given by Weber and Morris, implying that intraparticle diffusion (Step III) played a crucial role in the adsorption process (Figure S21, Supporting Information).^19^ Moreover, the two fitted lines did not pass through the origin, suggesting that adsorption was not merely controlled by Step III. It was worth to mention that the effect of transport in the solution (Step I) and film diffusion (Step II) were negligible because vigorous shaking was applied in the adsorption process to accelerate the transport process in bulk solution and to reduce the boundary layer thickness. Furthermore, in consideration that aptamers were needed to turn from inactive state into active structures before binding with their target molecules,^27^ the sorption step (Step IV) should be slower than Steps I and II. Therefore, it was reasonable to hypothesize that the adsorption rate was determined by the steps of intraparticle diffusion (Step III) and sorption on the active sites (Step IV).

The equilibrium adsorption isotherm of Hg^2+^ and BPA on the scavenger was fitted to the Langmuir model using the following equation

(3) $$\frac{C_{e}}{q_{e}}=\frac{C_{e}}{q_{0}}+\frac{1}{q_{0}b}$$

where *q*_e_ was the amount of adsorbed pollutants by sorbents at equilibrium (mg g^−1^), *C*_e_ was the equilibrium concentration of pollutants (mg L^−1^), *q*_0_ was the maximum adsorption (mg g^−1^), and *b* was the Langmuir constant (L mg^−1^). The equilibrium adsorption isotherm data of Hg^2+^ were fitted to this equation with a high correlation coefficient (0.9994), suggesting that monolayer adsorption of Hg^2+^ occurred on independent binding sites in the scavenger (Figure 5d). The maximum adsorption capacity of hydrogel scavenger for Hg^2+^ was calculated to be 0.71 mg g^−1^. Similarly, the equilibrium adsorption isotherm of BPA also exhibited typical Langmuir behavior with a high correlation coefficient (0.9987), and the maximum adsorption capacity of the scavenger toward BPA was determined to be 38.0 mg g^−1^ (Figure 5e). The above data indicated that this hydrogel scavenger also fulfills the features of rapid kinetics and high adsorption capacity in addition to perfect selectivity, multiple adsorbability, strong affinity, good reusability, and no secondary pollution. To conclude, the rationally designed hydrogel scavenger was demonstrated to well satisfy the main features of the idea adsorbents, showing its great potential for the next generation of water treatment system.

### 2.6 POU Device for Environmental Water Purification

**Figure 6:**
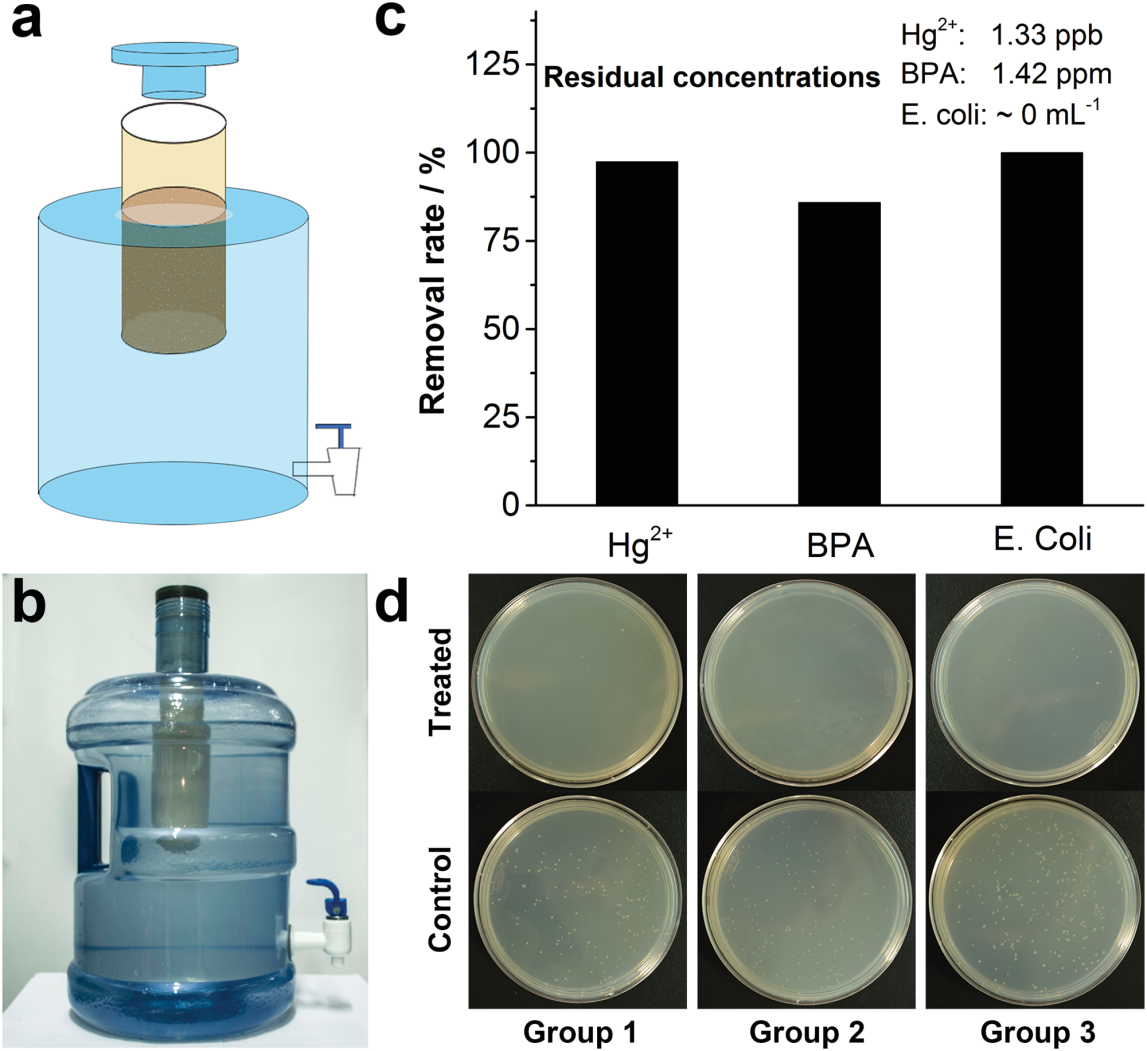
a) Schematic representation of the POU device. b) Photograph of the POU device. c) Removal efficiencies toward Hg^2+^, BPA, and *E. coli*. Insert: the residual concentrations of each pollutant. d) Photographs of *E. coli* culturing results. The upper row was the treated water and the lower row was the control samples.

Finally, a POU water purification device was further developed by packing the scavenger into an empty filter unit of a commercially available water purifier (**Figure** **6**a). Pollutants in aqueous solution can enter the scavenger to be captured by aptamers and silver ions diffused into the solution to kill pathogens. Figure 6b showed the photograph of the as‐prepared POU device. To investigate whether this device was able to remove pollutants from real environmental water, samples from the East Lake in Wuhan City were collected. No detectable Hg^2+^ and BPA was found in this water sample and trace amounts of the three pollutants were spiked into it. The as‐prepared environmental water sample was added into the POU device and the treated solution was collected to determine the residual contents of each pollutant. As shown in Figure 6c, Hg^2+^, BPA, and *E. coli* in this environmental water sample were efficiently removed by the POU device and the residual concentrations of Hg^2+^ and BPA were smaller than the drinking water norms. Figure 6d showed images of the cultured agar plates for *E. coli* in three groups of repeated tests, suggesting no obvious bacterial growth for samples after purification. These results clearly suggested the great potential of this hydrogel scavenger for practical water treatment applications.

## 3 Conclusions

In this work, we have highlighted the creation of a novel adsorbent by constructing a composite scavenger that featured highly porous structure allowing aptamers to form unique intricate binding pockets to capture pollutants, thereby affording perfect selectivity; anisotropic Janus nanoparticles functionalized with multiple aptamers to remove metal ions, organic pollutants, and pathogens simultaneously; aptamers well dispersed on the inner surface, thus providing strong binding affinity for pollutants; chemically stable aptamers with finely regulated secondary or tertiary structures facilitating good reusability; bifunctionalized nanoparticles incorporated into porous bulk structure to avoid secondary pollutant; open pore structure allowing for rapid diffusion of pollutants enable fast adsorption kinetics; and large surface area with highly accessible binding sites providing high adsorption capacity. The scavenger successfully conquered the contradiction between selectivity and multiple adsorbability, as well as the contradiction between binding affinity and reusability, thus demonstrating its great promise to serve as an ideal adsorbent. This adsorbent showed the outstanding performance in reducing Hg^2+^ concentration from 1 ppm to levels lower than 1.1 ppb; BPA from 60 ppm to level of smaller than 0.8 ppm, and can effectively kill more than 99.998% of *E. coli* at the same time. Moreover, a POU device was fabricated and it was capable of removing contaminants from environmental water with high efficiencies. The outstanding water purification performance of the scavenger demonstrated the promise of our design to be used for creating ideal adsorbents and the great potential of this scavenger to be applied to future water treatment systems. Furthermore, the strategy may be utilized to develop functional materials for both basic and applied research purposes, which can promote the development of materials science, environmental science, and biomedical science.

## 4 Experimental Section

*Synthesis of Fe_3_O_4_*–*Ag Janus Nanoparticles*: Fe_3_O_4_ nanoparticles were first synthesized according to a previously reported protocol.^28^ Typically, FeCl_3_·6H_2_O (2.7 g) and sodium oleate (9.125 g) were added into the mixture of ethanol (20 mL), *n*‐hexane (35 mL), and deionized water (15 mL). The mixture was allowed to react at 70 °C for 4 h under magnetic stirring and the obtained ferric oleate was washed at least three times with deionized water. The ferric oleate was completely dried in a vacuum oven before the following usage. Then, the dried ferric oleate (9.0 g) and sodium oleate (1.52 g) were dissolved in 1‐octadecene (25.0 g), and the solution was heated to 320 °C for 30 min in argon. The obtained black magnetite particles were magnetically separated and then washed with ethanol and *n*‐hexane for three times. The Fe_3_O_4_ particles (40 mg), silver nitrate (40.7 mg), oleylamine (0.5 mL), and 1,2‐dodecane glycol (0.1 g) were added into methylbenzene solution (20 mL). Then, the solution was heated to 110 °C under magnetic stirring for 1 h, and the obtained Fe_3_O_4_–Ag Janus nanoparticles were thoroughly washed with *n*‐hexane and ethanol for three times.

*Bifunctionalization of Fe_3_O_4_*–*Ag Janus Nanoparticles*: Fe_3_O_4_–Ag Janus nanoparticles were transferred from hydrophobic to hydrophilic by using dopamine‐ and thiol‐labeled Hg^2+^‐binding aptamers (designated as Apt‐Hg) as stabilizing ligands. Specifically, the as‐prepared hydrophobic Fe_3_O_4_–Ag Janus nanoparticles (10 mg) were added into water/ethanol solution (20 mL, V/V, 4:1) containing 2 μmol Apt‐Hg aptamers and 40 mg dopamine. The mixture was heated to 80 °C and allowed to react for 15 min under shaking. Then, the as‐obtained nanoparticles were magnetically isolated and washed with acetone and water for three times, respectively. The resultant Apt‐Hg modified nanoparticles were further immersed in 25% aqueous solution of glutaraldehyde overnight. After thoroughly washed with water, the nanoparticles were added into 20 mL solution containing BPA‐binding aptamers (designated as Apt‐BPA, 2 μmol) to react for another 10 h. Finally, the bifunctionalized nanoparticles were isolated with a permanent magnet and thoroughly washed with water for three times.

*Cyclical Removal of Multiple Pollutants with the Bifunctionalized Janus Nanoparticles*: Typically, 10 mg of biohybrids was dispersed in 5 mL aqueous solution containing Hg^2+^ (1 ppm) and BPA (60 ppm). The solution was allowed to react for 3 h under shaking. Then, the biohybrids were separated with a permanent magnet and the supernatant was collected for Hg^2+^ and BPA determination. Biohybrids were washed with HCl (pH 1) and deionized water for at least three times, respectively.^29^ Then, the resultant biohybrids were used for the next cycle. As for *E. coli*, the bacteria were incubated in Luria‐Bertani (LB) broth at 37 °C overnight and washed with deionized water for four times before the adsorption experiment. After that, 10 mg biohybrids were mixed with ≈10^5^ CFU mL^−1^ *E. coli* in 5 mL solution and were incubated for 3 h followed by magnetic separation of the biohybrids. The isolated adsorbents were washed with water for three times and used in the next cycle.

*Preparation of Hydrogel Scavenger*: Cellulose sample (0.8 g, 4 wt%) was dissolve in NaOH–urea aqueous solution under vigorously stirring. Epichlorohydrin was then dropped into the above cellulose solution and the mixture was stirred for another 2 h. After that, 60 mg bifunctionalized Fe_3_O_4_–Ag nanoparticles were added into 10 mL cellulose solution under stirring for 0.5 h to yield a homogeneous solution. Then, the solution was allowed to react at 37 °C for 10 h to form the composite hydrogel. The as‐prepared hydrogel was immersed in water for 2 d to get rid of the unreacted substances at 4 °C.

*Selectivity Tests*: Certain amounts of Hg(ClO_4_)·3H_2_O, BPA, NaNO_3_, KNO_3_, and Ca(NO_3_)_2_ were dissolved in water to from aqueous solution (Hg^2+^, 1 ppm; BPA, 60 ppm; Na^+^, 10 ppm; K^+^, 10 ppm; Ca^2+^, 10 ppm). Then, hydrogel scavenger was added to 30 mL of this water sample and the solution was allowed to react at 37 °C for 3 h under shaking. After that, the solution was collected to determine the residual contents of each substance.

*Multiple Adsorbability Tests*: In a typical experiment, water sample (30 mL) containing Hg^2+^ (1 ppm) and BPA (60 ppm) was treated with the scavenger for 3 h at 37 °C. The remaining Hg^2+^ was measured by atomic fluorescence spectrometry and BPA by HPLC. The removal of *E. coli* was performed with the following procedure. It was worth to mention that *E. coli* was treated alone to avoid uncontrollable effects on its growth activity caused by Hg^2+^ and BPA. Initially, *E. coli* was incubated in LB broth at 37 °C overnight. *E. coli* (≈10^5^ mL^−1^) was incubated with the scavenger for 3 h, then the supernatant was transferred to an LB agar plate for spread plate analysis. It was worth to stress that *E. coli* samples without treatment were diluted to ≈10^3^ mL^−1^ for the spread plate analysis. The number of the colonies was counted to determine the concentration of living *E. coli*. Another removal tests for trace amount of pollutants (Hg^2+^, 50 ppb; BPA, 10 ppm; *E. coli*, ≈10^3^ mL^−1^) were also conducted with the same procedure.

*Estimation of the Upper Limit of BPA in Drinking Water*: The recognized tolerable daily intake value of BPA was 0.05 mg g^−1^ body weight based on reports by European Food Safety Authority (EFSA).^30^ According to WHO's methods, the default assumption for water consumption by an adult should be 2 L d^−1^, and the body weight of an adult was assumed to be 60 kg.^25^ From these parameters, the upper limit of BPA in drinking water was easily determined to be 1.5 ppm.

*Cyclical Water Purification Experiments*: In a typical experiment, water samples (30 mL) containing Hg^2+^ (1 ppm) and BPA (60 ppm) were incubated with scavenger for 3 h under shaking. Then, the solution was collected to determine the residual concentrations of Hg^2+^ and BPA, and the scavenger was washed with HCl (pH 1) and water for three times, respectively. Afterward, the scavenger was put into the next cycle with the same procedure. The *E. coli* samples were treated alone with scavenger. Water samples containing *E. coli* (≈10^5^ mL^−1^) were incubated with hydrogel scavenger 3 h at 37 °C. Samples of supernatant were collected to determine the concentration of living *E. coli* by plaque assay techniques. The scavenger was washed with water and used for the next cycle.

*Adsorption Kinetics Study*: Water sample containing Hg^2+^ (1 ppm) and BPA (60 ppm) was incubated with hydrogel scavenger. At different time intervals, the supernatant was collected to determine the concentrations of each pollutant. Experimental data of Hg^2+^ and BPA adsorbed on hydrogel scavenger were fitted with the pseudo‐second‐order kinetic model.^19^

*Intraparticle Diffusion Study*: Intraparticle diffusion model was described by the equation given by Weber and Morris^19^

(4) $$q_{t}=K_{d}t^{1/2}+C$$

where *K*_d_ was the intraparticle diffusion rate constant (mmol g^−1^ min^−0.5^) and *C* was the thickness of the boundary layer.

*Equilibrium Adsorption Isotherm Study*: Water samples containing different amounts of Hg^2+^ and BPA were incubated with hydrogel scavenger for 3 h under shaking. Then, supernatant from each sample was collected for concentration determination.

*Purification of Environmental Water with POU Device*: The original adsorbents in the filter unit of a commercially available water purifier (BSP 0.9, Doulton) were removed. Then, the mixture of cellulose solution (300 mL) and bifunctionalized Janus nanoparticles (3.0 g) was transferred into the empty filter unit to form the hydrogel scavenger. Environmental water sample was filtered and disinfected before further use. Trace amounts of Hg^2+^ and BPA were spiked into the environmental water sample (Hg^2+^, 50 ppb; BPA, 10 ppm) and *E. coli* sample (≈10^3^ mL^−1^) was also prepared with environmental water. The filter unit with the scavenger was utilized to treat the above‐prepared contaminated water (7 L) and the solution was collected to determine the residual concentrations of each pollutant.

## Supporting information

As a service to our authors and readers, this journal provides supporting information supplied by the authors. Such materials are peer reviewed and may be re‐organized for online delivery, but are not copy‐edited or typeset. Technical support issues arising from supporting information (other than missing files) should be addressed to the authors.

Supplementary
