# Supplementary material for: A Targeted “Capture” and “Removal” Scavenger toward Multiple Pollutants for Water Remediation based on Molecular Recognition
Source: Adv Sci (Weinh). 2015 Dec 10;3(3):1500289. doi: 10.1002/advs.201500289 (PMC5064623; doi:10.1002/advs.201500289)
Supplement: Supplementary file 1 — Supplementary [file ADVS-3-1500289-s001.pdf]

Copyright WILEY-VCH Verlag GmbH & Co. KGaA, 69469 Weinheim, Germany, 2015.

ADVANCED SCIENCE
Open Access

Supporting Information

for *Adv. Sci.*, DOI: 10.1002/advs. 201500289

A Targeted "Capture" and "Removal" Scavenger toward Multiple Pollutants for Water Remediation based on Molecular Recognition

*Jie Wang, Haijing Shen, Xiaoxia Hu, Yan Li, Zhihao Li, Jinfan Xu, Xiufeng Song, Haibo Zeng, and Quan Yuan**

Supporting Information

A Targeted 'Capture' and 'Removal' Scavenger towards Multiple Pollutants for Water Remediation based on Molecular Recognition

*Jie Wang, Haijing Shen, Xiaoxia Hu, Yan Li, Zhihao Li, Jinfan Xu, Xiufeng Song, Haibo Zeng and Quan Yuan**

Chemicals

Cellulose sample (cotton linter pulp) was purchased from Hubei Chemical Fiber Co. Ltd (Xiangfan, China). Mercury perchlorate trihydrate ($Hg(ClO_4)_2{\cdot}3H_2O$) was provided by J&K Scientific. Bisphenol A (BPA, ≥99%), dopamine hydrochloride (98%) were purchased from Aladdin Reagents Co. Ltd (Shanghai, China). Ferric chloride hexahydrate ($FeCl_3{\cdot}6H_2O$, 99%), silver nitrate, sodium oleate ($C_{17}H_{33}CO_2Na$, CP), oleylamine, oleic acid (OA), 1-octadecene (ODE), epichlorohydrin (ECH), ethanol, hexane, toluene, glutaraldehyde (25%) were purchased from Sinopharm Chemical Reagent Co. (China).

Aptamers

Aptamers were all synthesized on an ABI3400 DNA/RNA synthesizer (Applied Biosystems, Foster City, CA, USA). The obtained sequences were purified by reversed-phase HPLC (Agilent, 1260 Infinity Quaternary, Japan) on a C-18 column. The 3'-S-S labeled aptamers was incubated with TCEP in 20 mM Tris/HCl (pH=7.4) buffer for 1 hour at room temperature to reduce disulfide bond to -SH groups. The obtained 3'-SH aptamers were further purified with a NAP-5 column.

Apt-Hg: 5'-CTT CTT TCT TCC CCT TGT TTG TTG CCC CCC CCC−SH-3'

Apt-BPA: 5'-CCG GTG GGT GGT CAG GTG GGA TAG CGT TCC GCG TAT GGC CCA GCG CAT CAC GGG TTC GCA CCA TTT TTT TTT-NH_2-3'

Instruments

The shape and size of Fe_3O_4-Ag nanoparticles were characterized with a transmission electron

microscope (TEM) with a working voltage of 200 kV (JEOL, JEM-2100, Japan); the high-angle annular dark-field scanning transmission electron microscopy and elemental mapping were obtained on a TEM (JEOL ARM200F) equipped with probe forming Cs corrector and Gatan image filter (Quantum 965). The structures of hydrogel was characterized on a field emission scanning electron microscope (Sigma， Zeiss， Germany) with an accelerating voltage of 10 kV. Mechanical properties of hydrogel scavenger were tested using a dynamic mechanical analyzer (DMA 2980 TA Instruments, Inc., USA).The crystal structure of Fe_3O_4-Ag was measured on an X-ray diffractometer (Bruker, D8 Advance, Germany) with a Cu-Kα radiation (λ = 1.5406 Å). The concentrations of all aptamers were measured by determining the absorption (260 nm) using a UV-Vis spectrometer (SHIMADZU, UV-2550, Japan). Zeta potential of all the samples were measured with a Zetasizer Nano ZS (Malvern). The concentrations of Hg^{2+} were determined by atomic fluorescence spectroscopy (Beijing Purkinje General Instrument Co., Ltd, PF6-2, China). The concentrations of Na^{+}, K^{+}, Ca^{2+} and $Fe^{2+/3+}$ were all determined with graphite furnace atomic absorption spectrometry. The concentrations of BPA was determined by HPLC (Agilent, 1100 series, Japan) equipped with a C-18 column (4.6 mm × 250 mm, 5 μm).

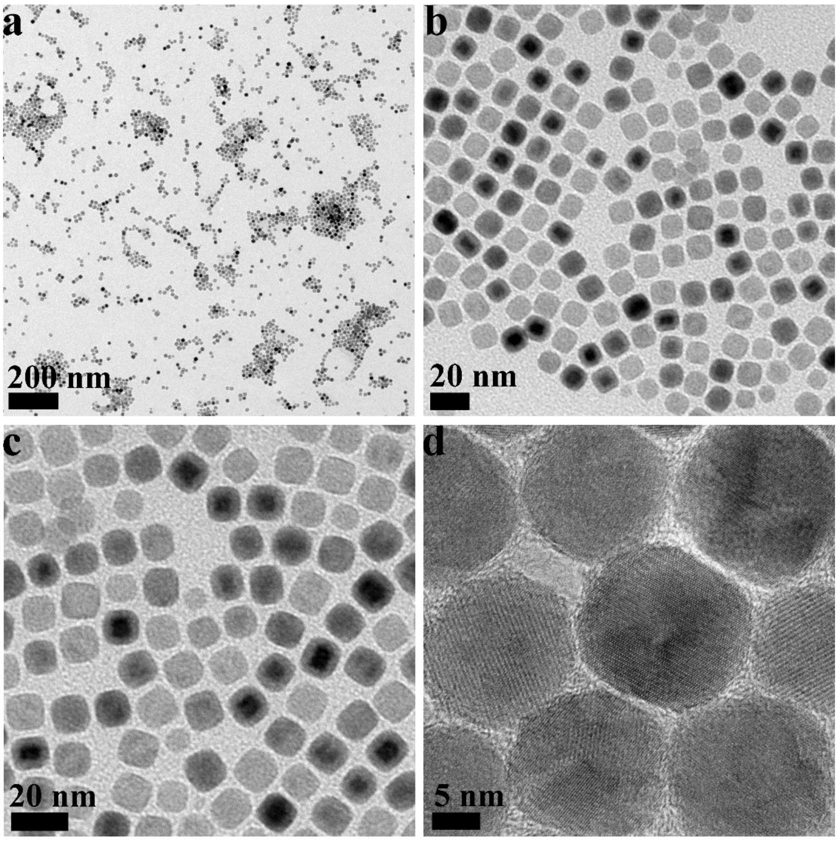

Figure S1. TEM images of Fe_3O_4 nanoparticles at different magnification.

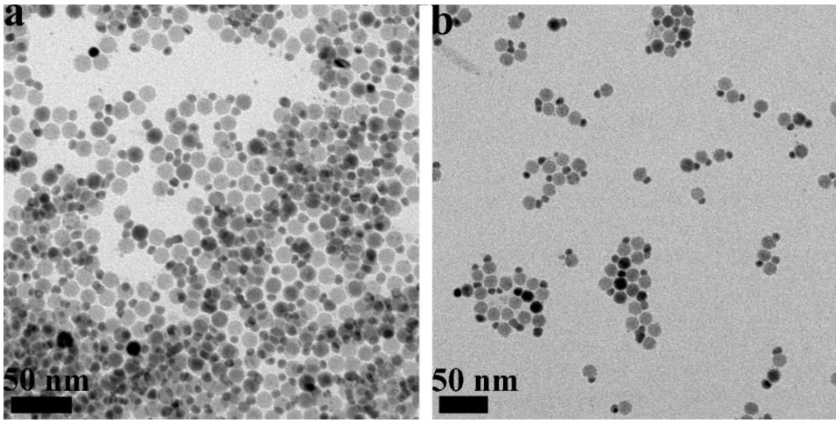

Figure S2. TEM images of Fe_3O_4-Ag nanoparticles at different magnification.

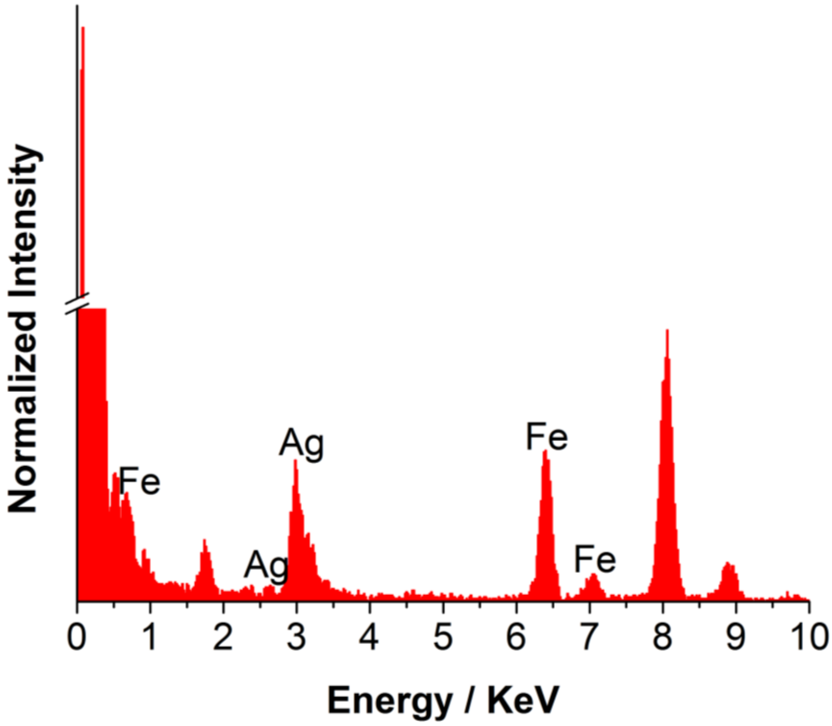

Figure S3. EDX analysis of Fe_3O_4-Ag nanoparticles.

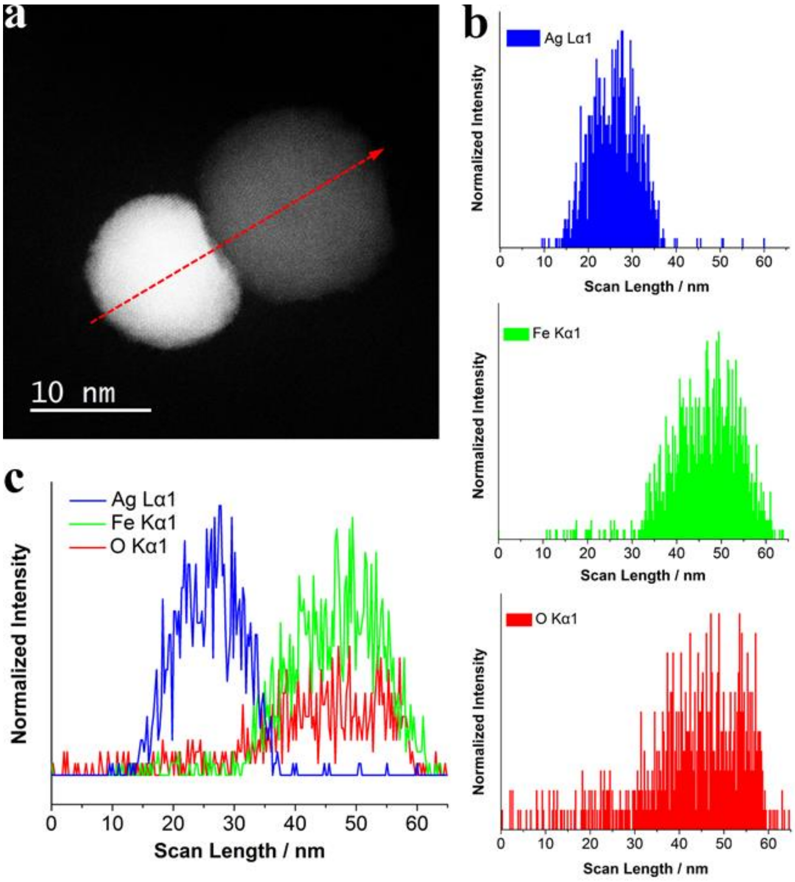

Figure S4. (a) HAADF-STEM image of a single Fe_3O_4-Ag nanoparticle with a highlighted red arrow showing the path of line scan. (b) Line scan spectral profiles of O (Kα), Fe (Kα) and Ag (Lα). (c) The corresponding merged map.

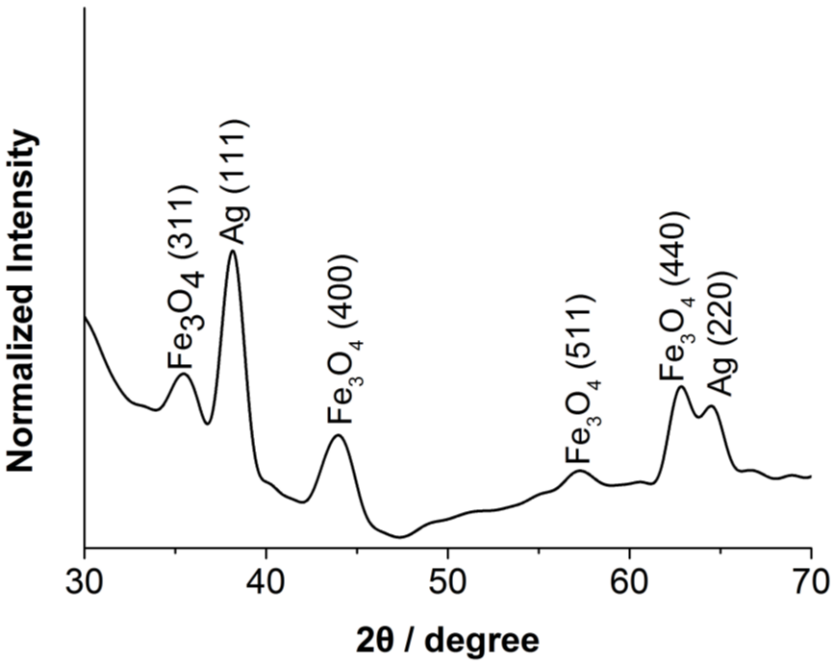

Figure S5. X-ray diffraction (XRD) pattern of the Fe_3O_4-Ag nanoparticles.

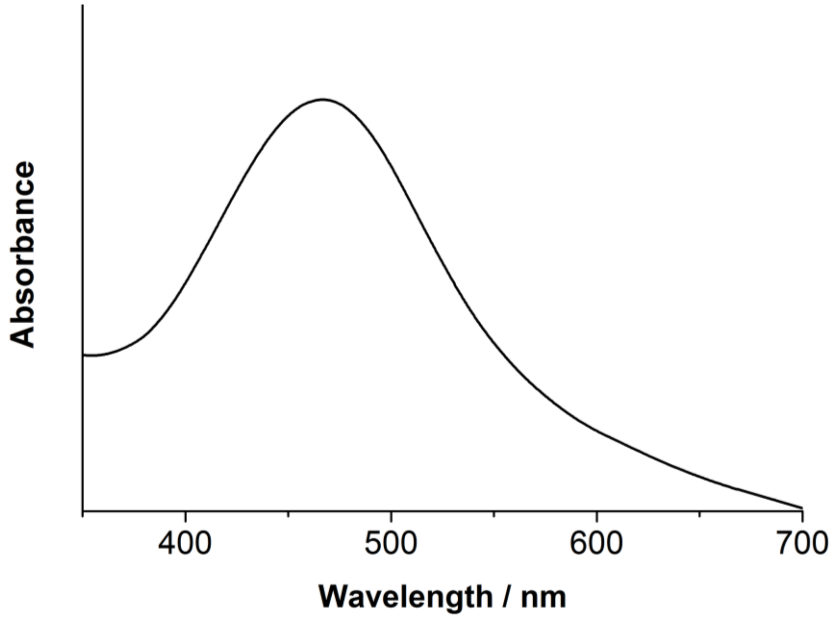

Figure S6. UV-Vis absorbance of the Fe_3O_4-Ag nanoparticles solution.

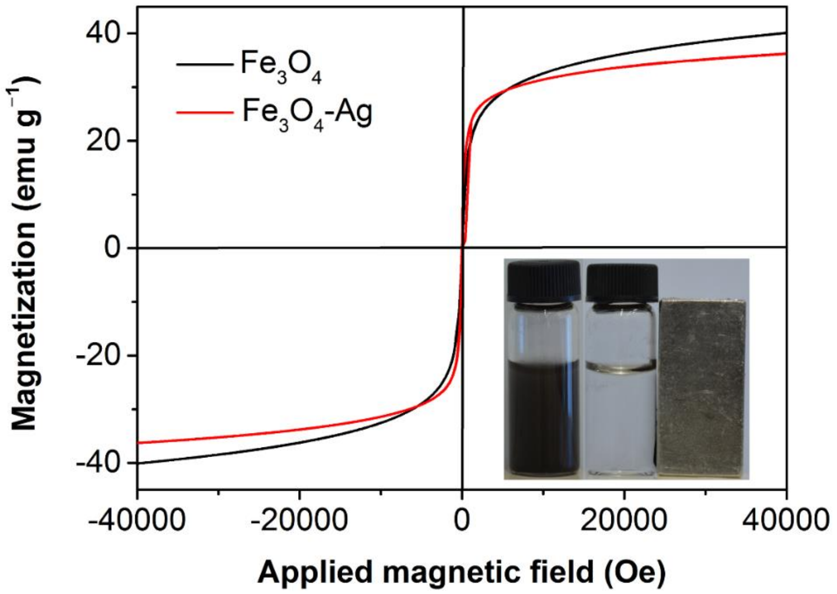

Figure S7. Field dependence of magnetization plot of Fe_3O_4 and Fe_3O_4-Ag nanoparticles at 300 K. Insert: aqueous dispersion of Fe_3O_4-Ag nanoparticles and the separation process with a permanent magnet.

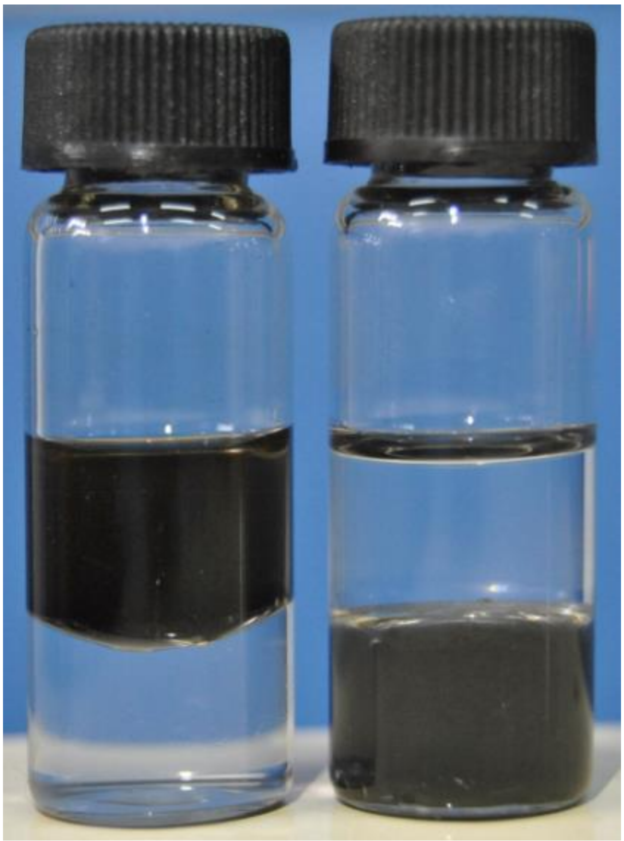

Figure S8. Photographs showing Fe_3O_4-Ag nanoparticles transformed from hydrophobic (left panel) to hydrophilic (right panel) with dopamine and Apt-Hg solution. The upper layer was toluene phase and the lower layer was water phase.

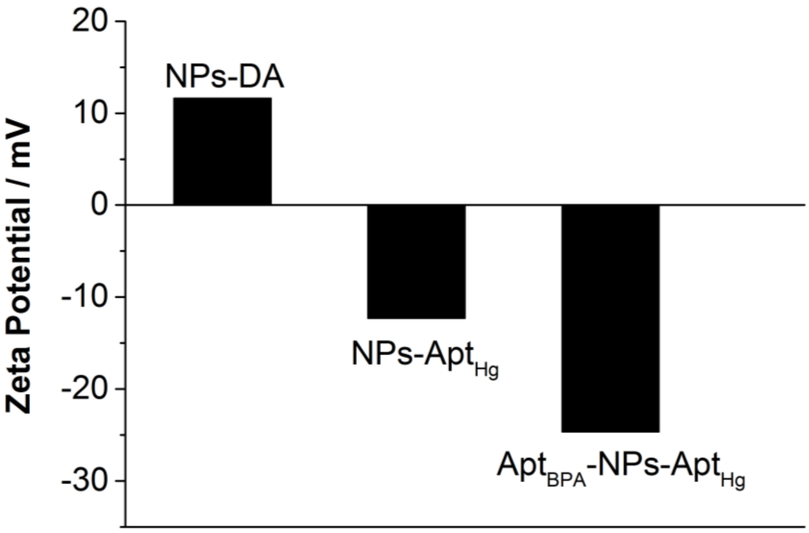

Figure S9. Zeta potential measurement for the functionalization process.

The bifunctionalized Janus nanoparticles were employed to treat aqueous solution containing Hg^{2+} and BPA first to preliminarily test its feasibility. As shown in Fig. S10 and S11, the as prepared bio-hybrids displayed ultra-high effectiveness in removing these two pollutants (Hg^{2+}, >99.93%; BPA, >98.7%) simultaneously and it can be recycled for 10 times without significant loss of sorption capabilities. Besides, Escherichia coli (E. coli) can also be effectively killed (>99.997%) by the bio-hybrids in all of the tested cycles (Fig. S12).

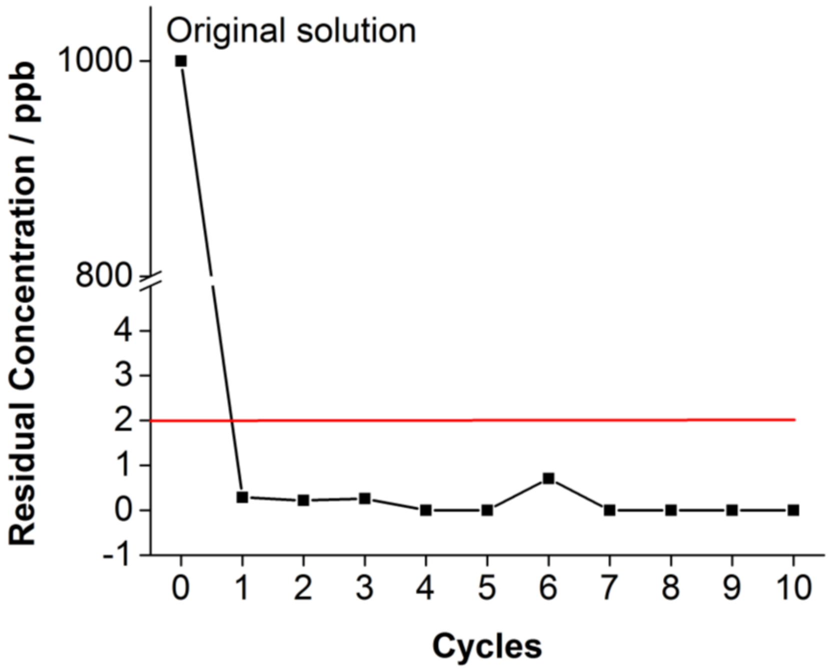

Figure S10. Cyclic removal efficiencies of bifunctionalized Janus nanoparticles towards Hg^{2+}. The red line showed the upper limit of concentration of Hg^{2+} in drinking water.

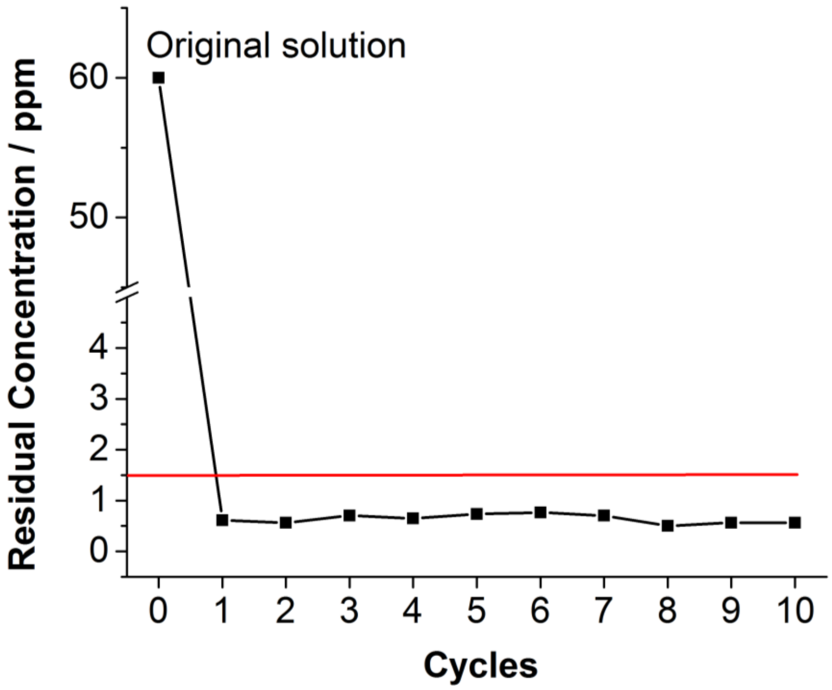

Figure S11. Cyclic removal efficiencies of bifunctionalized Janus nanoparticles towards BPA. The red line showed the upper limit of concentration of BPA in drinking water.

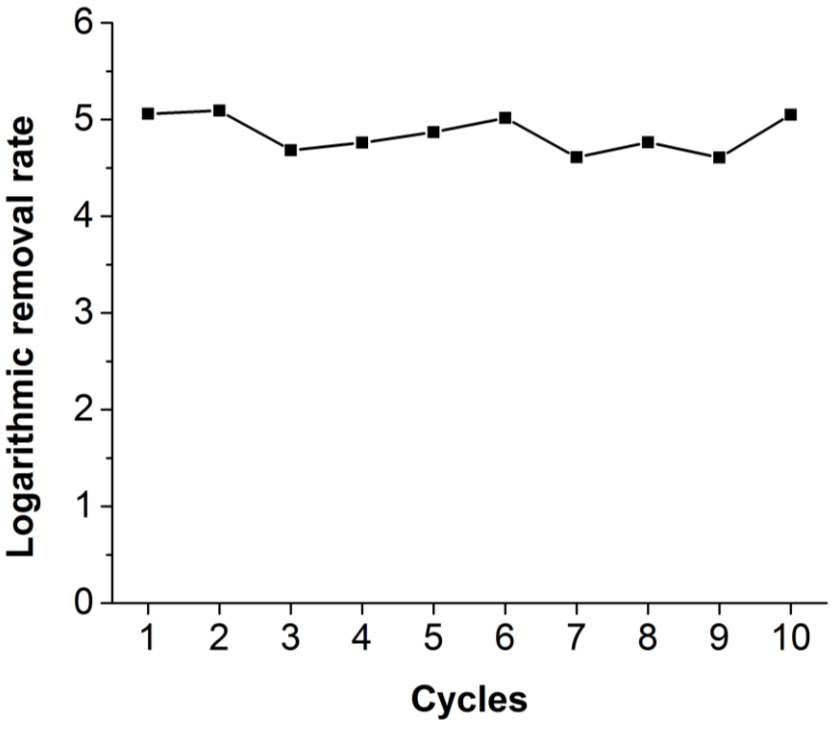

Figure S12. Cyclic disinfection efficiencies of bifunctionalized Janus nanoparticles towards E. coli.

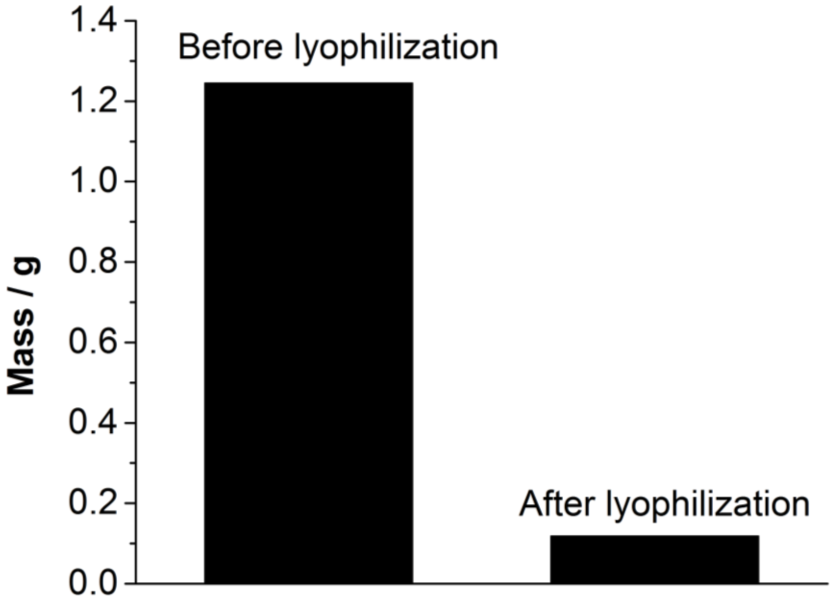

Figure S13. Water content measurement for the hydrogel scavenger, the water content was determined to be 90.49%.

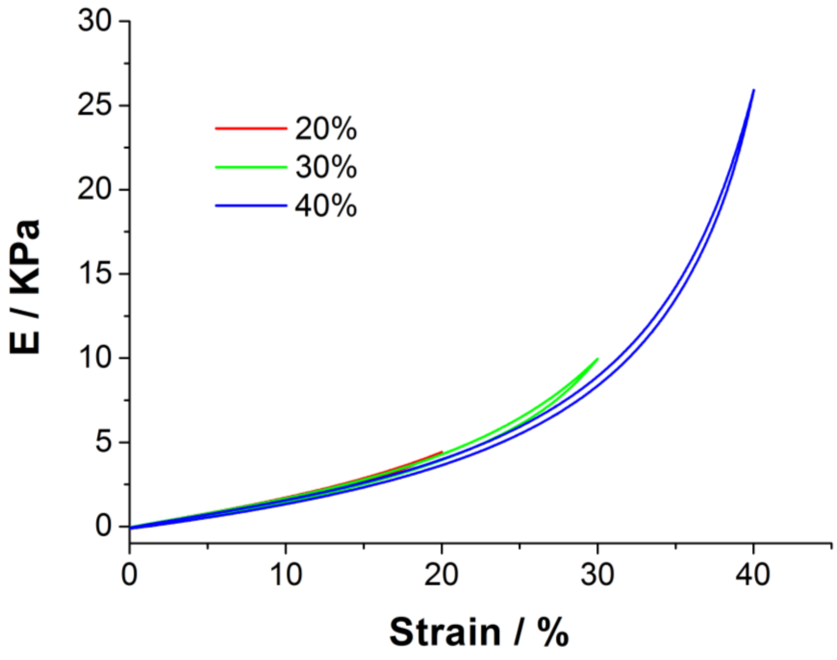

Figure S14. Stress–strain curves of the scavenger at strains of 20%, 30%, and 40%, respectively.

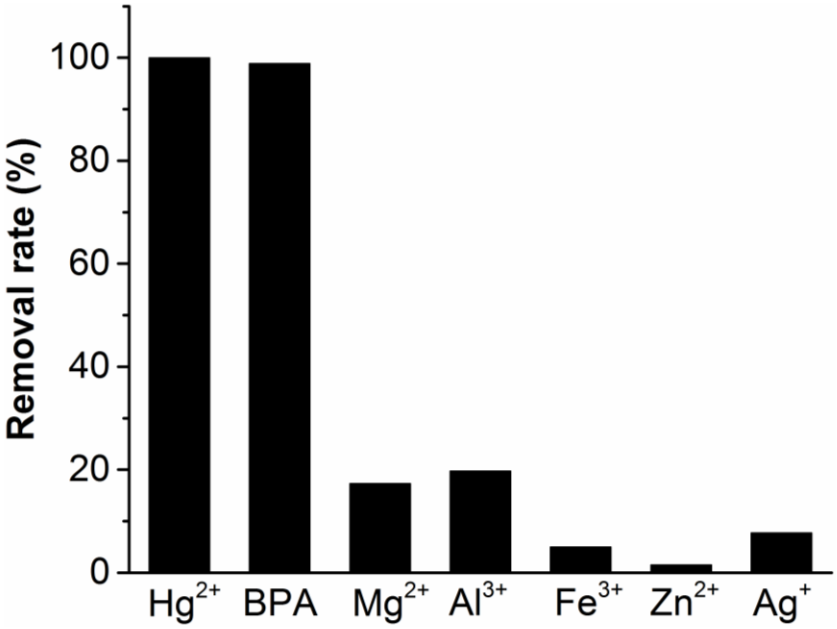

Figure S15. Removal efficiencies of the scavenger towards different compounds in the aqueous solution.

The variation in the concentrations of Hg^{2+}, BPA and E. coli in the presence of composite scavenger as a function of time was investigated. It was suggested that the residual concentrations of Hg^{2+} in the aqueous solution decreased sharply within the first 20 min (Figure S16), revealing that Hg^{2+} was rapidly captured by the scavenger. After that, it took another 80 min for the adsorption of Hg^{2+} onto the scavenger to reach the adsorption equilibrium. As for BPA, much slower adsorption kinetics was observed (Figure S17). The residual concentrations of BPA decreased gradually and reached a plateau at about 120 min. Figure S18 showed that nearly all of the E. coli was inactivated within 60 min, and essentially no bacterial growth was observed for the aqueous solution after exposed to the scavenger for 80 min.

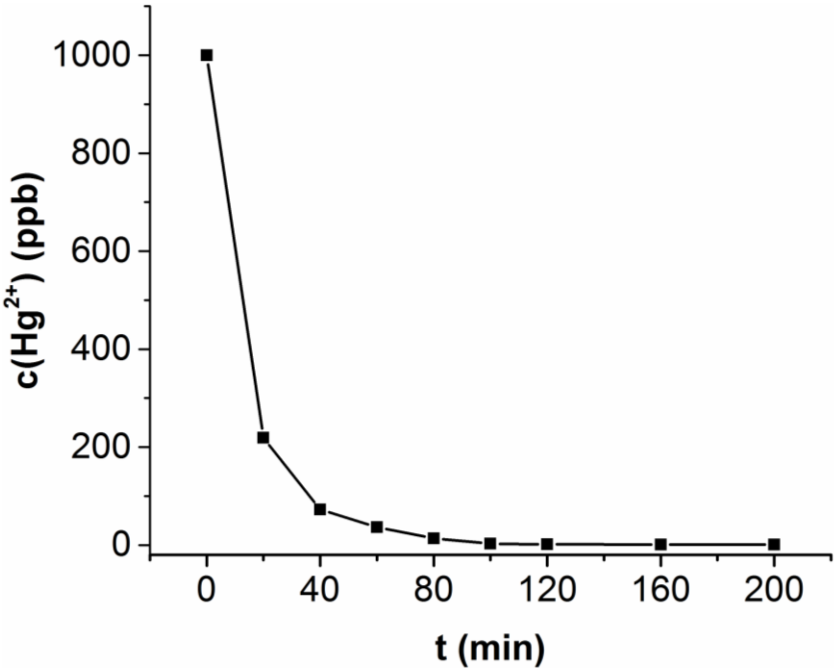

Figure S16. Time-evolution of the residual concentrations of Hg^{2+} in the solution.

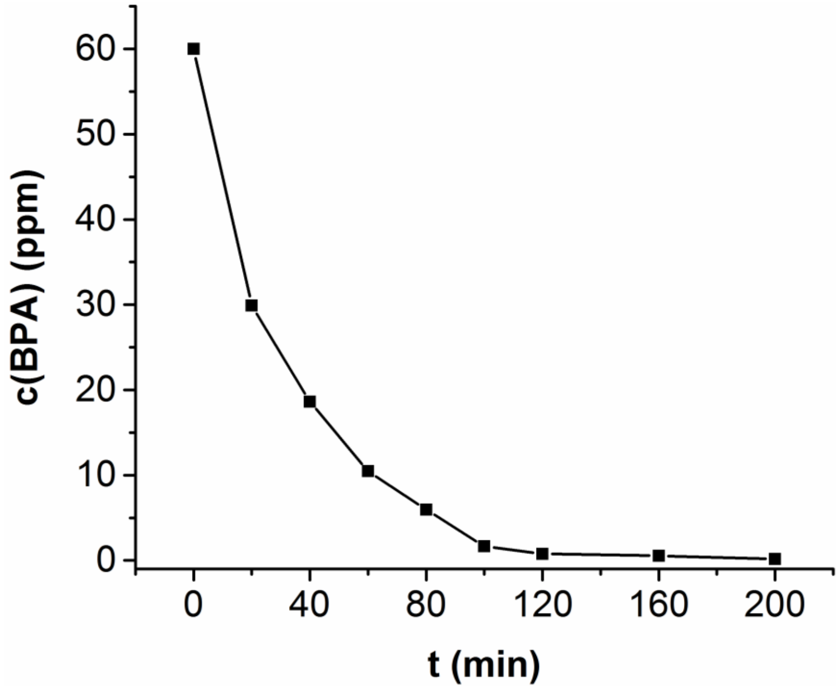

Figure S17. Time-evolution of the residual concentrations of BPA in the solution.

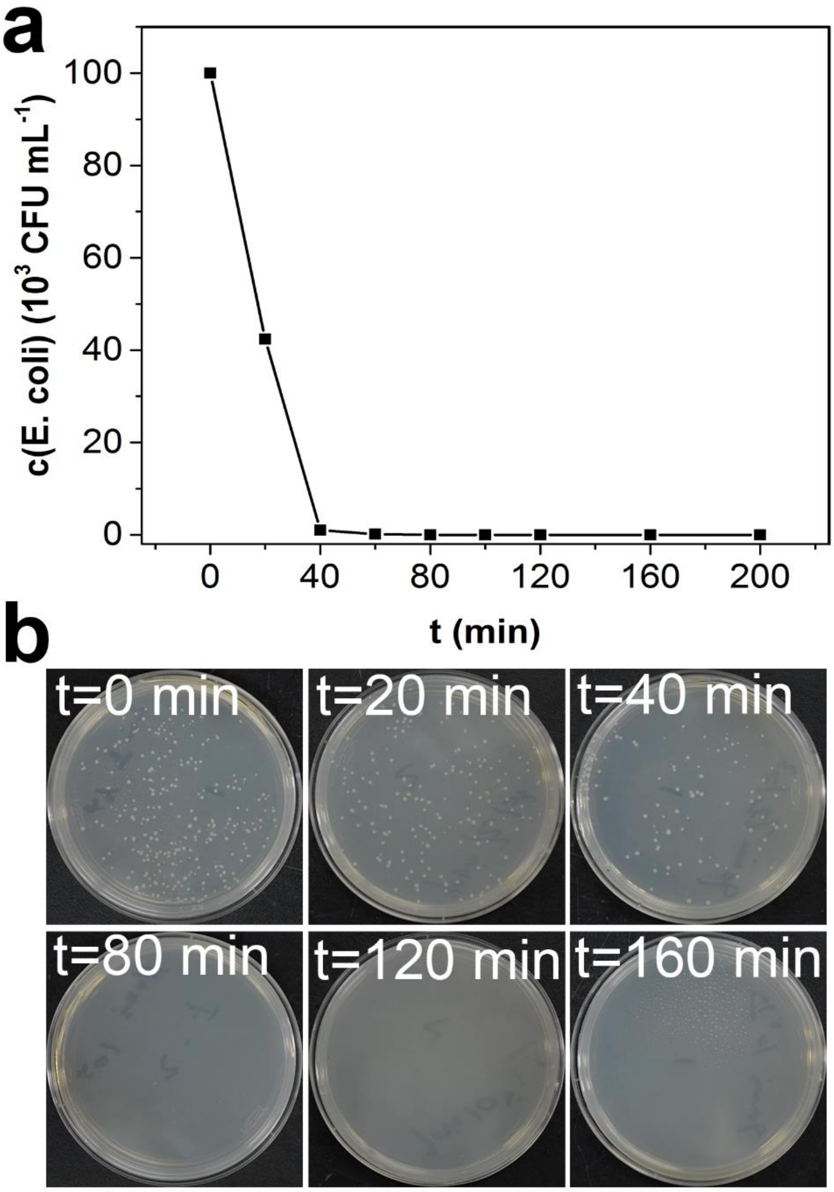

Figure S18. Time-evolution of the residual concentrations of E. coli in the solution.

Water samples with trace amounts of three main kinds of pollutants were also purified with ultra-high efficiencies by the scavenger (Figure S19), showing the great flexibility of this adsorbent for dealing with water with either high or low concentrations of pollutants.

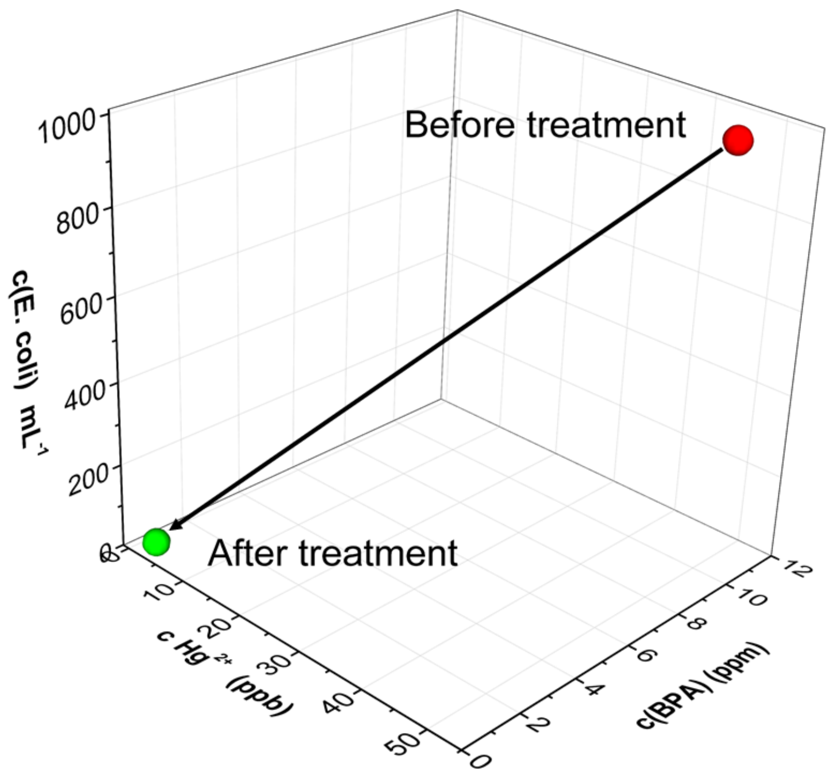

Figure S19. Removal of trace amounts of Hg^{2+} (50 ppb), BPA (10 ppm) and E. coli (10^3 mL^{-1}) with the scavenger from contaminated water.

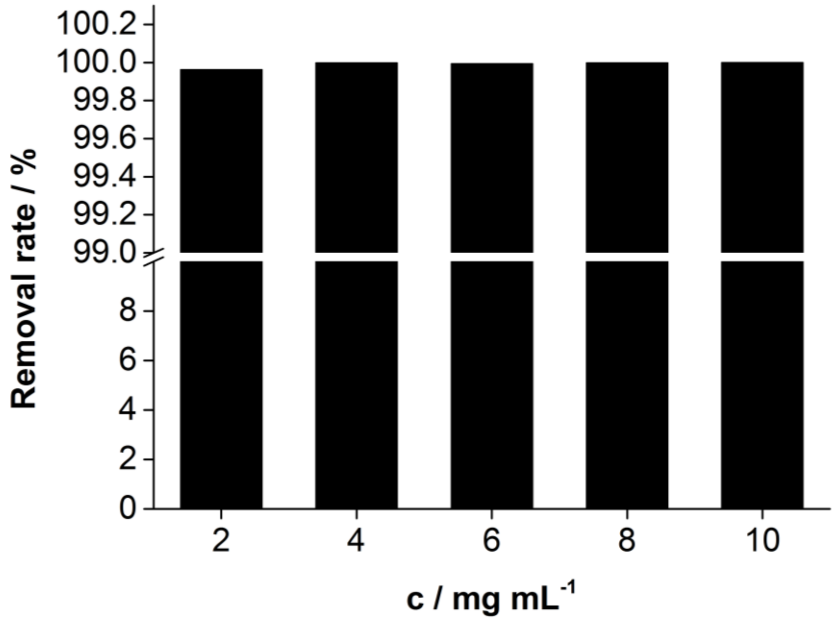

Figure S20. The disinfection efficiencies with different amounts of scavenger.

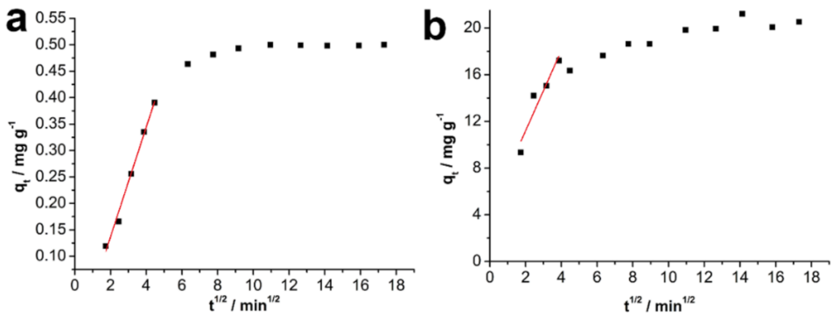

Figure S21. Application of the intraparticle diffusion model for the adsorption of Hg^{2+} (a) and BPA (b) on the hydrogel scavenger.

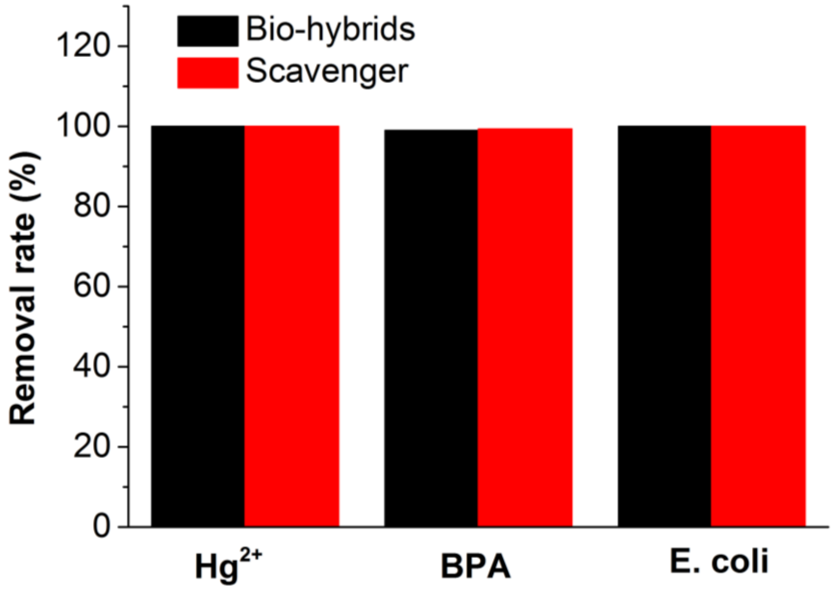

Figure S22. Comparison of the removal efficiencies between bifunctionalized Janus nanoparticles and the hydrogel scavenger.
